# Predictive factors of intracranial bleeding in head trauma patients receiving antiplatelet therapy admitted to an emergency department

**DOI:** 10.1186/s13049-018-0515-0

**Published:** 2018-06-19

**Authors:** Farès Moustafa, Jean Roubin, Bruno Pereira, Alain Barres, Jennifer Saint-Denis, Christophe Perrier, Marine Mondet, Frederic Dutheil, Jeannot Schmidt

**Affiliations:** 1 Emergency Department, Clermont-Ferrand University Hospital, Clermont-Ferrand, France; 2 Université Clermont Auvergne, Clermont-Ferrand, France; 3 Biostatistics Unit, DRCI, Clermont-Ferrand University Hospital, Clermont-Ferrand, France; 4 Department of Medical Information, University Hospital of Clermont-Ferrand, Clermont-Ferrand, France; 5 School of Exercise Science, Australian Catholic University, Melbourne, VIC Australia; 6 UMR CNRS 6024, “Physiological and Psychosocial Stress” Team, LAPSCO, Clermont-Ferrand, France; 7 Service des Urgences, CHU Clermont-Ferrand, 58 rue Montalembert, F-63003 Clermont-Ferrand, Cedex 1 France

## Abstract

**Background:**

In head trauma cases involving antiplatelet agent treatment, the French Society of Emergency Medicine recommends performing computed tomography (CT) scans to detect brain lesions, 90% of which are normal. The value of CT is still debatable given the scarce number of studies and controversial results.

**Methods:**

We used the RATED registry (Registry of patient with Antithrombotic agents admitted to an Emergency Department, NCT02706080) to assess factors of cerebral bleeding related to antiplatelet agents following head trauma.

**Results:**

From January 2014 to December 2015, 993 patients receiving antiplatelet agents were recruited, 293 (29.5%) of whom underwent CT scans for brain trauma. Intracranial bleeding was found in 26 (8.9%). Multivariate analysis revealed these patients more likely to have a history of severe hemorrhage (odds ratio [OR]: 8.47, 95% confidence interval [CI]: 1.56–45.82), dual antiplatelet therapy (OR: 6.46, 95%CI:1.46–28.44), headache or vomiting (OR: 4.27, 95%CI: 1.44–2.60), and abnormal Glasgow coma scale (OR: 8.60; 95%CI: 2.85–25.99) compared to those without intracranial bleeding. The predictive model derived from these variables achieved 98.9% specificity and a negative predictive value of 92%. The area under the ROC curve (AUROC) was 0.85 (95%CI: 0.77–0.93).

**Conclusions:**

Our study demonstrated that the absence of history of severe hemorrhage, dual antiplatelet therapy, headache or vomiting, and abnormal Glasgow coma scale score appears to predict normal CT scan following traumatic brain injury in patients taking antiplatelets. This finding requires confirmation by prospective studies.

**Trial registration:**

ClinicalTrials.gov number: NCT02706080.

## Background

Traumatic brain injury (TBI) is very common in emergency departments (EDs), with an annual incidence of approximately 150 to 300 per 100,000 persons in Europe, and one million per year in the USA [1–3]. Most (95%) are caused by mild head injury defined by a Glasgow coma scale score ≥ 13 [4, 5]. Moreover, TBI is a common cause of death and disability, most often in young people but increasingly among the elderly [6]. Patients over 65 years old represent 25% of all trauma-related deaths [7]. Falls are particularly the leading cause of death by trauma [8].

This population is especially cause for concern due to their increased use of antiplatelets. Some studies reported no correlation between acetylsalicylic acid (ASA) administration and the incidence of post-traumatic intracranial lesions [9, 10]. Others, however, have found a link between antiplatelet agents and intracranial hemorrhage or mortality [11–15]. Particularly, clopidogrel appears to be associated with an increased risk of morbidity [16, 17]. Recently, a meta-analysis revealed a correlation between antiplatelet therapy and post-traumatic cerebral hemorrhage (odds ratio [OR]: 1.87; 95% confidence interval [CI]: 1.27–2.74) [18].

Clinical prediction rules based on prospective studies can identify which head trauma patients are at low risk of intracerebral hemorrhage (ICH) or neurosurgical lesions [19, 20]. However, these studies excluded patients receiving antiplatelet agents. The French guidelines thus prioritize CT as the gold standard for head trauma detection during antiplatelet agent administration [21]. For the other guidelines, however, antiplatelet agents are not listed as ICH risk factors [22, 23]. Some authors have attempted to define predictive factors for cerebral bleeding, like the Glasgow coma scale, loss of consciousness, headache or vomiting, yet most included patients taking anticoagulants or antiplatelets [14, 15, 24].

Choosing the optimum way to assess head trauma in antiplatelet agent cases is a significant challenge facing emergency physicians. Given the small number of studies producing controversial findings, a specific study on the bleeding risk factors for these particular patients with head trauma appears crucial in order to avoid unnecessary CT scanning. Our study sought to assess the factors related to traumatic intracranial bleeding in patients taking antiplatelet agents admitted to our ED.

## Methods

### Inclusion criteria

Consecutive patients admitted to the ED of a university hospital and receiving antithrombotic treatment at the time of admission were logged in the RATED registry (Registry of patient with Antithrombotic agents admitted to an Emergency Department, NCT02706080). All patients (or their legal power of attorney) were informed of the potential use of their personal data and none opposed consent. This analysis was approved by the appropriate regional French research ethics committee (CPP Sud-Est VI, IRB number: 00008526–2013/CE37).

RATED is a monocentric, ongoing (from January 2014), observational registry of consecutive patients taking antithrombotic drugs, admitted to our University Hospital ED for any reason. As far as they were able, the emergency physicians enrolled consecutive patients during each patient’s medical management. Thus, data was recorded in a digital case report form in the hospital patient records.

### Study design

We conducted a monocentric, retrospective study that used prospectively-collected data from consecutive patients enrolled in the RATED registry from January 2014 to December 2015. For this study, to follow the French Society recommendations, all patients receiving antiplatelet drugs on admission, and presenting with head traumas received a CT scan within 4 to 8 h and were included [21]. Those under anticoagulants were excluded.

This study sought to assess the clinical predictive factors for intracranial bleeding in patients who underwent TBI while taking antiplatelets.

### Baseline variables

The following parameters are routinely recorded in RATED: patient’s baseline characteristics; clinical status including any coexisting or underlying conditions; bleeding risk factors described in the literature (age, previous stroke, previous gastrointestinal bleeding, renal impairment, anemia, thrombocytopenia, liver disease, cancer, hypertension, dementia, alcohol) [25–28], and the use of antiplatelet therapy; use of CT scan or ultrasound; laboratory data at baseline; the antithrombotic treatment (indication, time duration, drugs, doses); concomitant drugs; death during hospitalization. For this study, we focused on the intracranial bleeding predictive factors that are already known: age, history of major bleeding (history of bleeding leading to transfusion, bleeding in a critical area and bleeding leading to hemodynamic instability by taking into account the patient history during the different hospital stay), anemia (defined as a hemoglobin count < 12 g/dl in women and < 13 in men), thrombocytopenia (defined as a platelet count < 150 G/l), renal failure, alcohol intake, neurological examination, Glasgow coma scale, headache or vomiting, loss of consciousness, and amnesia [14, 21, 24, 25]. Radiological severity was evaluated by calculating the Rotterdam CT score on the first scan performed in the emergency room as follows: (a) status of basal cisterns subdivided into normal (0), compressed (1), or absent (2); (b) midline shift subdivided into 0-5 mm (0) or > 5 mm (1); (c) epidural hematoma subdivided into present (0) or absent (1); (d) traumatic subarachnoid hemorrhage or intraventricular hemorrhage subdivided into absent (0) or present (1) [29, 30]. According to the CT scan results, we then compared patients with intracranial bleeding (CT group+) to those without (CT group-).

### Statistical analysis

It was difficult to estimate a sample size based on the literature in order to identify predictive factors for intracranial bleeding in patients taking antiplatelets presenting with head trauma. While numerous rules-of-thumb have previously been suggested for determining the minimum number of subjects required for conducting multiple regression analyses, these are heterogeneous and often have minimal empirical evidence. For multiple regression models, some authors suggest variable ratios of 15:1 or 30:1 when generalization is critical [31–34]. Considering these works and the intracranial bleeding rate, we deemed a sample size of approximately 300 subjects relevant to obtain satisfactory statistical power.

All statistical analyzes were performed with Stata software (Version 13, StataCorp, College Station, US) for a two-sided error significance level of 5%. Continuous data was presented as mean ± standard deviation or median [interquartile range], according to statistical distribution (Shapiro-Wilk test for normality). Comparisons between groups (CT group- and CT group+) were performed using classic statistical tests: Student’s t-test or the nonparametric Mann-Whitney test if the t-test assumptions were not met ([i] normality and [ii] homoscedasticity, analyzed by Fisher-Snedecor). For the categorical parameters, groups were compared using Chi-squared test or Fisher’s exact test. Multivariable logistic regression analysis was then performed considering covariates determined according to univariate results (*p* < 0.20) [35] and clinical relevance (following the literature) like age, gender, and loss of consciousness. A particular attention has been paid to the study of multicollinearity and interactions between covariates 1) studying the relationships between the covariables and 2) evaluating the impact to add or delete variables on multivariable model. The selection model was carried out by backward stepwise strategy based on Akaike information criteria. Then, the final model was validated by a two-step bootstrapping process. For each step, bootstrap samples with replacements (*n* = 1000) were generated from the training set. In the first phase, the percentage of models including each initial variable was determined by the classic stepwise approach. Then, in the second phase, the parameters of generalized linear regression (logistic for dichotomous dependent variables) of the final model were independently estimated. Finally, the bootstrap estimates associated with each covariate regression coefficient, along with their associated standard errors, were averaged from replicates. The results were expressed as odds-ratios (OR) with 95% confidence intervals (95%CI). To illustrate these results, a receiver operating characteristic (ROC) analysis was proposed, with the area under the curve (AUROC) estimated and presented with 95%CI.

## Results

**Fig. 1:**
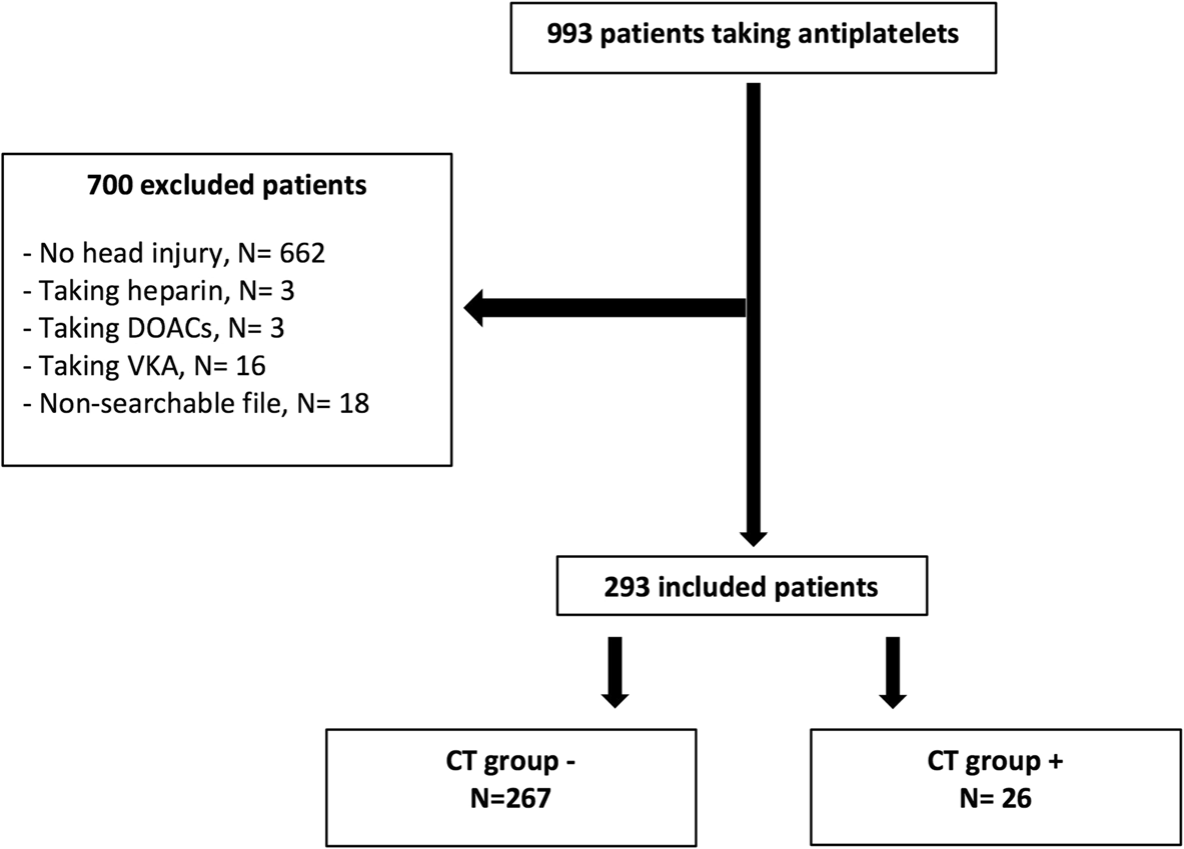
Flowchart. Abbreviations: DOACs: direct oral anticoagulants; VKA: vitamin K antagonist; CT: computed tomography

From January 2014 to December 2015, of over 993 patients taking antiplatelet drugs, 293 (29.5%) patients presenting with TBI and CT scans were included (Fig. 1). Of these, CT scan revealed no intracranial bleeding in 267 (91.1%) (CT group-), versus bleeding in 26 (8.9%) (CT group+). Overall, 262 (89.4%) were injured by falls, 22 (7.5%) by road accidents, two (0.7%) by assaults, and seven (2.4%) by other causes.

Mean age, patient > 75 years, and female-male rate were similar between the CT group- and CT group+ (Table 1). However, patients in the CT group+ were more likely to have major bleeding history and concomitant therapy with ASA and clopidrel than those in the CT group- (15% vs. 3 and 19% vs. 6%, respectively). Following their TBI, those in the CT group+ were more likely to present with loss of consciousness or amnesia and headaches or vomiting than those in the CT group- (73% vs. 38.5, 34.6% vs. 9.7%, respectively). Moreover, on arrival, CT group+ patients exhibited lower median Glasgow Coma scales than the CT group- (14 vs.15).

**Table 1:** Characteristics of patients with traumatic brain injury according to presence or not of intracranial bleeding on CT scan

	CT group -	CT group +	*p*-value
Patient*, N*	*267*	*26*	
Sex			
Women, n (%)	145 (54%)	11 (52%)	0.24
Age, mean +/− sd	80+/−11.1	80+/−10.5	0.98
> 65 years, n (%)	237 (88%)	24 (92%)	0.75
> 75 years, n (%)	209 (78%)	19 (73%)	0.54
Medical history, n (%)			
Stroke	61 (22%)	8 (32%)	0.30
Cancer	36 (13%)	4 (15%)	0.76
Major bleeding	9 (3%)	4 (15%)	*0.02*
Risk of falling	149 (55%)	19 (73%)	0.10
Renal or hepatic failure	69 (25%)	5 (19%)	0.45
Hypertension	158 (63%)	18 (75%)	0.24
Antiplatelet, n (%)			
ASA alone	199 (74.5%)	17 (65.4%)	0.31
Clopidogrel alone	46 (17.2%)	4 (15.4%)	0.81
ASA + clopidogrel	18 (6%)	5 (19%)	*0.04*
ASA + anti-Gp2b3a	4 (1.5%)	0 (0%)	1
Clinical characteristics, n (%)			
High-energy trauma	35 (13%)	5 (19%)	0.37
Alcohol	13 (4%)	0 (0%)	0.61
Loss of consciousness or amnesia	103 (38.5%)	19 (73%)	*< 0.001*
Headache or vomiting	26 (9.7%)	9 (34.6%)	*< 0.001*
Glasgow coma scale, median (IQR)	15 [15–15]	14 [13–15]	*< 0.001*
Biological characteristics, n (%)			
Anemia	67 (27%)	8 (30%)	0.68
Thrombocytopenia	23 (9%)	2 (8%)	1
CrCl ml/min, median (IQR)	66 [51–84]	65 [52–77]	0.72

*CT* computed tomography, *ASA* acetylsalicylic acid, *CrCl* creatinine clearance, *IQR* interquartile range

Among the 26 patients in CT group+, the Rotterdam CT-score was 1 for 10 patients (38.5%), 2 for 10 (38.5%), 3 for three (11.5%), and 4 for three (11.5%). Thirteen patients had only one cerebral bleeding and the other 13 had multiple cranial bleeding. The CT scan found 16 (61.5%) subdural hematoma, 13 (50%) subarachnoid hemorrhage, 10 (38.5%) intraparenchymal hematoma and 0 extradural hematoma. Moreover, six patients (23.1%) underwent surgery (1 decompressive craniectomy, 1 external ventricular drainage and 4 drainage) and five (19.2%) died during hospitalization (median: 2.5 days, interquartile range [IQR] [1–11]:), despite two undergoing neurosurgery.

On multivariable analysis, patients with history of major bleeding (OR: 8.47; 95%CI: 1.56–45.82) receiving concomitant therapy with ASA and clopidogrel (OR: 6.46; 95%CI: 1.46–28.44), and presenting with headache or vomiting on arrival (OR: 4.27; 95%CI: 1.44–12.60), or abnormal Glasgow coma scale (OR: 8.60; 95%CI: 2.85–25.99) were at increased risk of intracranial bleeding following TBI if taking antiplatelets (Table 2).

**Table 2:** Multivariate analysis of patient characteristics according to the risk of intracranial bleeding on CT scan

	Odds ratio	95%CI	*p*-value
Major bleeding	8.47	1.56–45.82	0.013
ASA + clopidogrel	6.46	1.46–28.44	0.014
Headache or vomiting	4.27	1.44–12.60	0.008
Glasgow coma scale	8.60	2.85–25.99	< 0.001
Women	1.59	0.60–4.15	0.34
Age	0.98	0.94–1.03	0.63
Loss of consciousness or amnesia	2.21	0.81–6.02	0.11

*ASA* acetylsalicylic acid, *CI* confidence interval

**Fig. 2:**
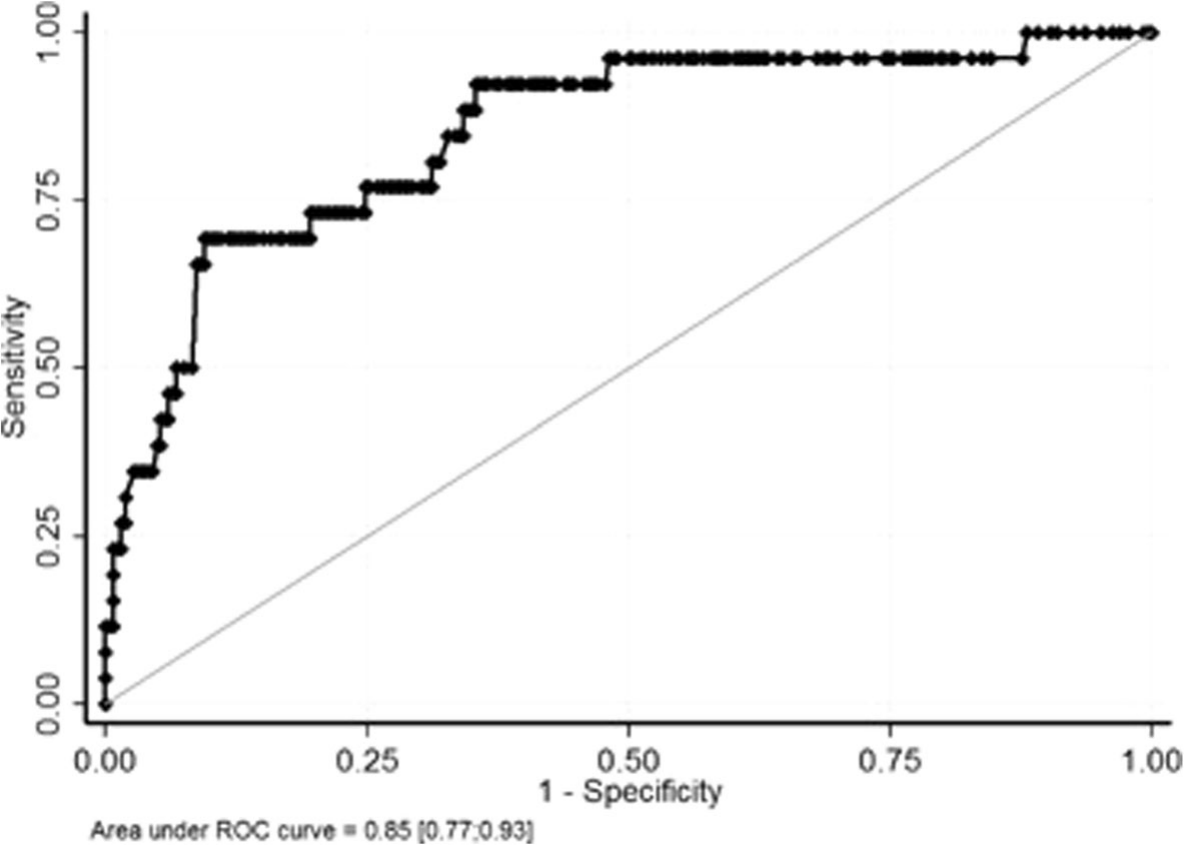
Receiver operating characteristic curve from final multivariate analysis. Abbreviations: ROC: receiver operating characteristic

The AUROC obtained from this multivariable analysis was high: 0.85 (95%CI 0.77–0.93). The Receiver Operating Characteristic (ROC) curve is shown in Fig. 2.

## Discussion

Our study reported on a large series of consecutive patients with TBI taking antiplatelet agents only, revealing that one in every 10 patients had intracranial bleeding. This result is consistent with reports of previous studies [24, 36, 37]. We found that four variables, easily available at baseline, may help clinicians identify patients with increased risk of intracranial bleeding: history of major bleeding, concomitant ASA with clopidogrel treatment, Glasgow coma scale, and headache or vomiting. Interestingly, the absence of all four of these factors on admission constitutes a predictive model with an area under the curve of 0.85 (95%CI: 0.77–0.93) to avoid intracranial bleeding.

Unexpectedly, after multivariable analysis, our study revealed that neither age nor loss of consciousness were predictive of intracranial bleeding. Age is a well-known bleeding risk factor, used in many bleeding risk scores [25, 28, 38, 39]. Nevertheless, our study along with others have demonstrated that old age, independent of signs and symptoms, is not considered an a priori risk factor for intracranial lesions, with 89% of our patients being over 65 years old [9, 40]. Previous studies suggested that patients who sustain minor head injuries are more likely to suffer intracranial bleeding if they have a history of loss of consciousness [13, 41]. However, as loss of consciousness was a subjective risk factor, other studies did not associate it with intracranial bleeding [42–45].

French guidelines recommend a CT scan 4 to 8 h following TBI in patients taking antiplatelet agents, whereas others do not constitute antiplatelet use as a risk factor of intracranial bleeding [21–23]. TBI cases with antiplatelet agents were associated with higher mortality compared to those without [46, 47]. The mechanism for this appears to be the extent of intracranial bleeding in patients taking antiplatelet agents, making them more likely to present more severe intracranial bleeding [46]. These severe intracranial bleeding cases involving antiplatelets were associated with 50% mortality, similar to the high mortality rate that occurred in those receiving anticoagulants [46, 48]. For patients receiving anticoagulants, bleeding progression was shown to be prevented and mortality reduced when these patients’ treatments were quickly reversed [49, 50]. However, for those taking antiplatelet agents, no reverse therapy exists.

A further challenge is posed by the increasing overcrowding of EDs by patients [51, 52]. This leads to long waiting times before a CT scan can be carried out, presenting a risk that patients needing urgent care may not be treated in time [53]. One recent study reported that a combination of clinical information upon ED admission enables early and more adequate risk stratification [54, 55]. Interestingly, to our knowledge, this is the first real-life study to find significant predictive factors of intracranial bleeding following TBI with antiplatelet treatment, with half the patients thus able to avoid CT scan.

Our study has potential limitations, however. First, since our study is an observational study (and not a randomized trial), our data is only hypothesis-generating, potentially providing a useful basis for future controlled clinical trials. Secondly, emergency physicians were free to choose whether or not to perform CT scans for TBI patients taking antiplatelet agents, and were probably more likely to avoid CT scans for patients at low risk of intracranial bleeding. Thirdly, the patients were recruited in a single center, which can cause less reproducibility, though avoids any variability of practices in different centers. Unfortunately, patients with normal CT scan weren’t follow after there discharged from the ward to look at delayed intracranial hemorrhage. However, it wasn’t recommended by French guidelines based on the study of Af-Geijerstam et al. who showed that, in a computed tomography strategy with a mean of 5.2 h after head trauma, no patients with normal findings on the scan had later complications (“false negatives”) [21, 56]. The main strengths of our observations were a real-life management of TBI under antiplatelet agents and a high number of consecutively included patients. Moreover, Considering the model proposed in this work, our results seem powerful and robust. According to works proposed by Tosteson et al. and Demidenko, the statistical power was greater than 85% [57, 58].

## Conclusion

Our study demonstrated that the absence of history of severe hemorrhage, dual antiplatelet therapy, headache or vomiting, or abnormal Glasgow coma scale score appears to predict a normal CT scan following TBI in patients taking antiplatelets. This finding do not apply to patients with anticoagulants and needs to be validated by prospective studies to avoid unnecessary CT scans being performed in this particular population presenting with TBI and taking antiplatelet agents admitted to EDs.

## Abbreviations

ASA: Acetylsalicylic acid
CI: Confidence interval
CrCl: Creatinine clearance
CT: Computed tomography
DOACs: Direct oral anticoagulants
ED: Emergency departments
ICH: Intracerebral hemorrhage
IQR: Interquartile range
OR: Odds Ratio
RATED: Registry of patient with Antithrombotic agents admitted to an Emergency Department
ROC: Receiver operating characteristic
TBI: Traumatic Brain Injury
VKA: Vitamin K antagonist.

## References

[1] Tagliaferri F, Compagnone C, Korsic M (2006). A systematic review of brain injury epidemiology in Europe. Acta Neurochir.

[2] Jager TE, Weiss HB, Coben JH, Pepe PE (2000). Traumatic brain injuries evaluated in U.S. emergency departments, 1992-1994. Acad Emerg Med.

[3] Rutland-Brown W, Langlois JA, Thomas KE, Xi YL (2006). Incidence of traumatic brain injury in the United States, 2003. J Head Trauma Rehabil.

[4] Fabbri A, Servadei F, Marchesini G (2010). Predicting intracranial lesions by antiplatelet agents in subjects with mild head injury. J Neurol Neurosurg Psychiatry.

[5] Cassidy JD, Carroll LJ, Peloso PM (2004). Incidence, risk factors and prevention of mild traumatic brain injury: results of the WHO collaborating Centre task force on mild traumatic brain injury. J Rehabil Med.

[6] Roozenbeek B, Maas AIR, Menon DK (2013). Changing patterns in the epidemiology of traumatic brain injury. Nat Rev Neurol.

[7] Gallagher SF, Williams B, Gomez C (2003). The role of cardiac morbidity in short- and long-term mortality in injured older patients who survive initial resuscitation. Am J Surg.

[8] Schwab CW, Kauder DR (1992). Trauma in the geriatric patient. Arch Surg.

[9] Spektor S, Agus S, Merkin V, Constantini S (2003). Low-dose aspirin prophylaxis and risk of intracranial hemorrhage in patients older than 60 years of age with mild or moderate head injury: a prospective study. J Neurosurg.

[10] Di Bartolomeo S, Marino M, Valent F, De Palma R (2014). Effects of anticoagulant and antiplatelet drugs on the risk for hospital admission for traumatic injuries: a case-control and population-based study. J Trauma Acute Care Surg.

[11] Siracuse JJ, Robich MP, Gautam S (2010). Antiplatelet agents, warfarin, and epidemic intracranial hemorrhage. Surgery.

[12] Batchelor JS, Grayson A (2013). A meta-analysis to determine the eff ect of anticoagulation on mortality in patients with blunt head trauma. Br J Neurosurg.

[13] Brewer ES, Reznikov B, Liberman RF (2011). Incidence and predictors of intracranial hemorrhage after minor head trauma in patients taking anticoagulant and antiplatelet medication. J Trauma.

[14] Reddy S, Sharma R, Grotts J (2014). Incidence of intracranial hemorrhage and outcomes after ground-level falls in geriatric trauma patients taking preinjury anticoagulants and antiplatelet agents. Am Surg.

[15] Fabbri A, Servadei F, Marchesini G (2013). Antiplatelet therapy and the outcome of subjects with intracranial injury: the Italian SIMEU study. Crit Care.

[16] Jones K, Sharp C, Mangram AJ, Dunn EL (2006). The effects of preinjury clopidogrel use on older trauma patients with head injuries. Am J Surg.

[17] Levine M, Wyler B, Lovecchio F (2014). Risk of intracranial injury after minor head trauma in patients with pre-injury use of clopidogrel. Am J Emerg Med.

[18] van den Brand CL, Tolido T, Rambach AH (2017). Systematic review and meta-analysis: is pre-injury antiplatelet therapy associated with traumatic intracranial hemorrhage?. J Neurotrauma.

[19] Stiell IG, Wells GA, Vandemheen K (2001). The Canadian CT head rule for patients with minor head injury. Lancet.

[20] Smits M, Dippel DWJ, Steyerberg EW (2007). Predicting intracranial traumatic findings on computed tomography in patients with minor head injury: the CHIP prediction rule. Ann Intern Med.

[21] Jehlé E, Honnart D, Grasleguen C (2012). Minor head injury (Glasgow Coma Score 13 to 15): triage, assessment, investigation and early management of minor head injury in infants, children and adults. Ann Fr Med Urgence.

[22] Jagoda AS, Bazarian JJ, Bruns JJ (2008). Clinical policy: neuroimaging and Decisionmaking in adult mild traumatic brain injury in the acute setting. Ann Emerg Med.

[23] Servadei F, Teasdale G, Merry G (2001). Defining acute mild head injury in adults: a proposal based on prognostic factors, diagnosis, and management. J Neurotrauma.

[24] Nishijima DK, Offerman SR, Ballard DW (2013). Risk of traumatic intracranial hemorrhage in patients with head injury and preinjury warfarin or clopidogrel use. Acad Emerg Med.

[25] Gage BF, Yan Y, Milligan PE (2006). Clinical classification schemes for predicting hemorrhage: results from the National Registry of atrial fibrillation (NRAF). Am Heart J.

[26] Kearon C, Ginsberg JS, Kovacs MJ (2003). Comparison of low-intensity warfarin therapy with conventional-intensity warfarin therapy for long-term prevention of recurrent venous thromboembolism. N Engl J Med.

[27] Palareti G, Cosmi B (2009). Bleeding with anticoagulation therapy - who is at risk, and how best to identify such patients. Thromb Haemost.

[28] Pisters R, Lane DA, Nieuwlaat R (2010). A novel user-friendly score (HAS-BLED) to assess 1-year risk of major bleeding in patients with atrial fibrillation: the euro heart survey. Chest.

[29] Huang YH, Deng YH, Lee TC, Chen WF (2012). Rotterdam computed tomography score as a prognosticator in head-injured patients undergoing decompressive craniectomy. Neurosurgery.

[30] Maas AIR, Hukkelhoven CWPM, Marshall LF, Steyerberg EW (2005). Prediction of outcome in traumatic brain injury with computed tomographic characteristics: a comparison between the computed tomographic classification and combinations of computed tomographic predictors. Neurosurgery.

[31] Green SB (1991). How many subjects does it take to do a regression analysis. Multivariate Behav Res.

[32] Pedhazur EJ (1997) Multiple regression in behavioral research : explanation and prediction. 10.2307/2285468.

[33] Harris RJ. A primer of multivariate statistics; 3rd ed. Taylor and Francis; 2014. http://cds.cern.ch/record/1974897.

[34] Hair JFJ, William CB, Babin BJ, Anderson RE (2010). Multivariate data analysis; A global perspective.

[35] Steyerberg EW, Eijkemans MJ, Harrell FE, Habbema JD (2000). Prognostic modelling with logistic regression analysis: a comparison of selection and estimation methods in small data sets. Stat Med.

[36] Undén J, Romner B (2010). Can low serum levels of S100B predict normal CT findings after minor head injury in adults?: an evidence-based review and meta-analysis. J Head Trauma Rehabil.

[37] Thaler HW, Schmidsfeld J, Pusch M (2015). Evaluation of S100B in the diagnosis of suspected intracranial hemorrhage after minor head injury in patients who are receiving platelet aggregation inhibitors and in patients 65 years of age and older. J Neurosurg.

[38] Fang MC, Go AS, Chang Y (2011). A new risk scheme to predict warfarin-associated hemorrhage: the ATRIA (anticoagulation and risk factors in atrial fibrillation) study. J Am Coll Cardiol.

[39] O’Brien EC, Simon DN, Thomas LE (2015). The ORBIT bleeding score: a simple bedside score to assess bleeding risk in atrial fibrillation. Eur Heart J.

[40] Fabbri A, Servadei F, Marchesini G (2005). Clinical performance of NICE recommendations versus NCWFNS proposal in patients with mild head injury. J Neurotrauma.

[41] Hamden K, Agresti D, Jeanmonod R (2014). Characteristics of elderly fall patients with baseline mental status: high-risk features for intracranial injury. Am J Emerg Med.

[42] Gómez PA, Lobato RD, Ortega JM, De La Cruz J (1996). Mild head injury: differences in prognosis among patients with a Glasgow coma scale score of 13 to 15 and analysis of factors associated with abnormal CT findings. Br J Neurosurg.

[43] Falimirski ME, Gonzalez R, Rodriguez A, Wilberger J (2003). The need for head computed tomography in patients sustaining loss of consciousness after mild head injury. J Trauma.

[44] Türedi S, Hasanbasoglu A, Gunduz A, Yandi M (2008). Clinical decision instruments for CT scan in minor head trauma. J Emerg Med.

[45] Ganetsky M, Lopez G, Coreanu T, et al. Risk of intracranial hemorrhage in ground-level fall with antiplatelet or anticoagulant agents. Acad Emerg Med. 2017:1–9. 10.1111/acem.13217. 10.1111/acem.13217 28475282

[46] F a I, G a H, Junn FS (2008). Predictors of mortality in trauma patients with intracranial hemorrhage on preinjury aspirin or clopidogrel. J Trauma.

[47] Ohm C, Mina A, Howells G (2005). Effects of antiplatelet agents on outcomes for elderly patients with traumatic intracranial hemorrhage. J Trauma.

[48] Mina AA, Knipfer JF, Park DY (2002). Intracranial complications of preinjury anticoagulation in trauma patients with head injury. J Trauma.

[49] Tazarourte K, Riou B, Tremey B (2014). Guideline-concordant administration of prothrombin complex concentrate and vitamin K is associated with decreased mortality in patients with severe bleeding under vitamin K antagonist treatment (EPAHK study). Crit Care.

[50] Ivascu FA, Howells GA, Junn FS, et al (2005) Rapid warfarin reversal in anticoagulated patients with traumatic intracranial hemorrhage reduces hemorrhage progression and mortality. J Trauma 59:1131–1137-1139. 10.1097/01.ta.0000189067.16368.83 10.1097/01.ta.0000189067.16368.83 16385291

[51] McCaig LF, Burt CW (2004). National Hospital Ambulatory Medical Care Survey: 2002 emergency department summary. Adv Data..

[52] Burt CW, McCaig LF, Rechtsteiner EA (2007). Ambulatory medical care utilization estimates for 2005. Adv Data..

[53] Ruan S, Noyes K, Bazarian JJ (2009). The economic impact of S-100B as a pre-head CT screening test on emergency department management of adult patients with mild traumatic brain injury. J Neurotrauma.

[54] Schuetz P, Hausfater P, Amin D (2013). Optimizing triage and hospitalization in adult general medical emergency patients: the triage project. BMC Emerg Med.

[55] Schuetz P, Hausfater P, Amin D (2015). Biomarkers from distinct biological pathways improve early risk stratification in medical emergency patients: the multinational, prospective, observational TRIAGE study. Crit Care.

[56] Af Geijerstam J-L, Oredsson S, Britton M Medical outcome after immediate computed tomography or admission for observation in patients with mild head injury: randomised controlled trial. 10.1136/bmj.38918.669317.4F. 10.1136/bmj.38918.669317.4F PMC1557917 16895944

[57] Tosteson TD, Buzas JS, Demidenko E, Karagas M (2003). Power and sample size calculations for generalized regression models with covariate measurement error. Stat Med.

[58] Demidenko E (2007). Sample size determination for logistic regression revisited. Stat Med.

